# Temporal Analysis of the Microbial Community from the Crystallizer Ponds in Cabo Rojo, Puerto Rico, Using Metagenomics

**DOI:** 10.3390/genes10060422

**Published:** 2019-05-31

**Authors:** Ricardo L. Couto-Rodríguez, Rafael Montalvo-Rodríguez

**Affiliations:** 1 Department of Microbiology and Cell Science, University of Florida, Gainesville, FL 32603, USA; r.coutorodriguez@ufl.edu; 2 Biology Department, Box 9000, University of Puerto Rico, Mayagüez, PR 00681, USA

**Keywords:** metagenomics, hypersaline, halophilic archaea, Puerto Rico, Caribbean

## Abstract

The Cabo Rojo solar salterns are a hypersaline environment located in a tropical climate, where conditions remain stable throughout the year. These conditions can favor the establishment of steady microbial communities. Little is known about the microbial composition that thrives in hypersaline environments in the tropics. The main goal of this study was to assess the microbial diversity present in the crystallizer ponds of Cabo Rojo, in terms of structure and metabolic processes across time using metagenomic techniques. Three samplings (December 2014, March and July 2016) were carried out, where water samples (50 L each) were filtered through a Millipore pressurized filtering system. DNA was subsequently extracted using physical–chemical methods and sequenced using paired end Illumina technologies. The sequencing effort produced three paired end libraries with a total of 111,816,040 reads, that were subsequently assembled into three metagenomes. Out of the phyla detected, the microbial diversity was dominated in all three samples by *Euryarchaeota,* followed by *Bacteroidetes* and *Proteobacteria.* However, sample MFF1 (for Muestreo Final Fraternidad) exhibited a higher diversity, with 12 prokaryotic phyla detected at 34% NaCl (w/v), when compared to samples MFF2 and MFF3, which only exhibited three phyla. Precipitation events might be one of the contributing factors to the change in the microbial community composition through time. Diversity at genus level revealed a more stable community structure, with an overwhelming dominance of the square archaeon *Haloquadratum* in the three metagenomes. Furthermore, functional annotation was carried out in order to detect genes related to metabolic processes, such as carbon, nitrogen, and sulfur cycles. The presence of gene sequences related to nitrogen fixation, ammonia oxidation, sulfate reduction, sulfur oxidation, and phosphate solubilization were detected. Through binning methods, four putative novel genomes were obtained, including a possible novel genus belonging to the *Bacteroidetes* and possible new species for the genera *Natronomonas, Halomicrobium*, and *Haloquadratum*. Using a metagenomic approach, a 3-year study has been performed in a Caribbean hypersaline environment. When compared to other salterns around the world, the Cabo Rojo salterns harbor a similar community composition, which is stable through time. Moreover, an analysis of gene composition highlights the importance of the microbial community in the biogeochemical cycles at hypersaline environments.

## 1. Introduction

Marine solar salterns are classified as hypersaline environments due to their high NaCl concentrations (above 3 M) [1]. The organisms that frequently predominate in these ecosystems are known as halophiles, which can thrive at around 10–15% NaCl (w/v) or above [2]. Halophiles possess a wide variety of applications, including enzymes used for food processing and biosynthetic processes like hydrolases, such as amylases, lipases, and proteases [3,4,5]. Hypersaline habitats have been extensively studied worldwide, especially in template locations such as Turkey, where the microbial diversity was determined from six hypersaline lakes across the country; the Santa Pola salterns in Alicante, Spain (which have been the most extensively studied); and the Dead Sea [6,7,8,9,10]. The extreme conditions of these environments make them model ecosystems for understanding microbial community dynamics [11]. Despite these findings, relatively few studies have been performed in tropical environments, where conditions normally remain relatively stable throughout the year, with temperatures ranging from 35 to 40 °C year-round, as well as low precipitation rates and no drastic weather events in seasons (fall, winter, autumn, summer). Drastic changes can be introduced by hurricanes (like hurricane Maria, in September 2017) but this phenomenon does not happen every year.

The Cabo Rojo solar salterns are a tropical hypersaline environment that have been the subject of numerous diversity studies and novel microbes have been described. *Halogeometricum borinquense* was first isolated from these salterns [12] and subsequent microbial diversity surveys from the crystallizers and surrounding areas (mainly *Avicennia germinans* forests) yielded additional novel organisms (*Haloterrigena thermotolerans*, *Halomonas avicenneae* (now *Kushneria avicennia*e), *Halobacillus mangrovii*, *Kushneria aurantia*, as well as two recent novel isolates proposed as *‘Haloarcula rubripromontorii’* and *‘Halorubrum tropicale’*) [13,14,15,16,17,18]. These findings suggest a more diverse community than that reported in other solar salterns worldwide. However, the aforementioned studies were performed using culture-dependent methods, where approximately 0.1% of the diversity can be isolated in pure culture [19]. Metagenomics has emerged as an answer to these limitations and has been applied for studies in habitats with high salinity. The most predominant studies have been undertaken in the Santa Pola salterns in Alicante, Spain, where the overwhelming dominance of the square archaeon, *Haloquadratum walsbyi,* has been confirmed at salinities of 30% NaCl (w/v) and above, as well as the presence of novel microbial groups previously undescribed in these environments [7,8]. Furthermore, ecological processes have also been described, using metagenomic techniques in the Santa Pola salterns. Other studies around the world include the Lake Tyrell, where the novel group of *Nanohaloarchaea* was first detected, and the Atacama Desert, where metagenomic analysis of endolithic halite microbial communities from the Salar Grande returned a novel genome also belonging to the *Nanohaloarchaea* [20,21]. These studies have shown that metagenomic methods in hypersaline environments successfully provide more comprehensive answers to community composition, as well as possible functions within these communities. The discovery of novel microbial groups has shifted our understanding of hypersaline environments towards new directions. This development will continue as new techniques become available and more data are released, which will in turn provide more tools towards characterizing novel taxa, as well as novel biocatalysts.

In this study, the aim was to obtain a comprehensive assessment of microbial and functional gene diversity at the crystallizer ponds of the solar salterns in Cabo Rojo, by performing a temporal metagenomic analysis. With this information, we intend to establish comparisons in terms of functional and microbial diversity with other metagenomes available from solar salterns around the world. The microbial diversity in the Cabo Rojo salterns was determined by means of culture independent methods, by using the pyrosequencing of partial 16S rRNA genes from a previous study [22]. However, to our knowledge, a full scale temporal metagenomic approach to assess gene diversity has not yet been performed in an extreme environment in the Caribbean. 

## 2. Materials and Methods

### 2.1. Sampling and DNA Purification

**Figure 1:**
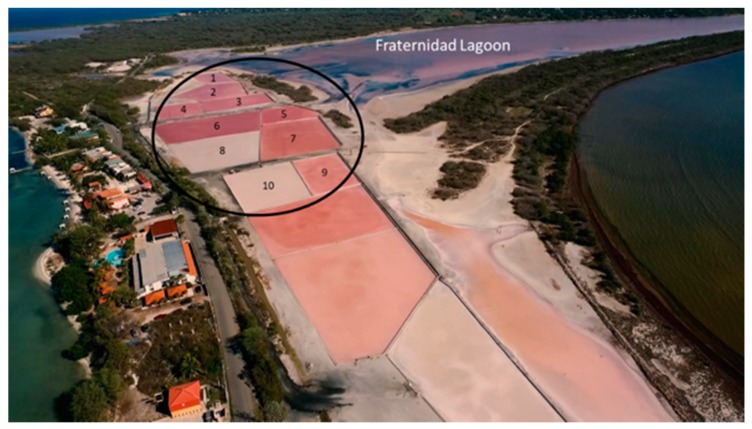
Aerial map of the Solar Salterns of Cabo Rojo (17°57′25.2″ N, 67°11′58.0″ W). Water samples (5 L) were obtained from ten crystallizers (circled in black) and pooled together into one sample (50 L). Three samplings were performed on the same crystallizers in rainy (December 2014), and dry (March and July 2016) seasons. Photo taken by “Puerto Rico desde el Aire” reproduced with permission.

Samplings (50 L per sample) were carried out in months of rainy (December 2014), and dry (March and July 2016) seasons. Water samples were taken from the crystallizer ponds at the Cabo Rojo salterns (17°57′25.2″ N, 67°11′58.0″ W). This crystallizer system is served by the hypersaline Fraternidad Lagoon (Figure 1). Five-liter samples (from between the surface to 10 cm depth) of ten crystallizers were taken (Figure 1) and pooled to obtain a 50 L total volume per sampling. Samples were named MFF1 for the first sampling, MFF2 for the second sampling, and MFF3 for the third sampling (MFF stands for “Muestra Final Fraternidad”, which means Fraternidad Final Sample). Temperature and salinity were taken for each sample (using a Fisherbrand^TM^ salinity refractometer, Fisher Scientific, Pittsburgh, PA, USA) and averaged. Samples were then transported back to the laboratory. Each 50 L sample of saltern water was differentially filtered using a Millipore® pressurized filtering system, consisting of two nitrocellulose membrane filters (EMD Millipore, Burlington, MA, USA) of different pore sizes. The first membrane possessed a pore size of 5.0 µm, which was intended to retain eukaryotic cells, whereas the second membrane, with a pore size of 0.22 µm, was used for the collection of prokaryotic cells. Metagenomic DNA extraction was performed on cells present on the 0.22 µm membrane using the physical–chemical methods described previously by Martín-Cuadrado et al. [23]. Concentration and purity of DNA were measured using a Nanodrop^TM^ spectrophotometer (Thermo Scientific, Waltham, MA, USA). Furthermore, a 0.8% (w/v) agarose gel electrophoresis was carried out in order to corroborate DNA quality before sequencing. Metagenomic DNA was then stored at −20 °C until it was used for sequencing.

### 2.2. DNA Sequencing and Metagenome Assembly

DNA sequencing was performed using an Illumina HiSeq 2500. Library preparation and sequencing was carried out by the Molecular Research DNA (MR DNA) facility in Shallowater, TX. The sequencing reads obtained were quality checked using FastQC [24]. Low quality reads were trimmed for assembly using BBDuk (Geneious, Newark, NJ, USA). Taxonomy was assigned by comparing raw reads to Ribosomal Database Project (RDP)using a minimum alignment length of 100 bp and a threshold of 97%. Afterwards, assembly of remaining reads was performed using a MetaSPAdes assembler [25], where the quality of metagenomes assemblies was compared based on N50 values (median length of contigs), total contigs obtained, as well as the largest contig. Taxonomic and functional annotation of the assembled metagenome was carried out using the MG-RAST [26] pipeline, which aligned sequences to reference databases, such as the KEGG, eggNOG, COG, and SEED subsystems [27,28,29].

### 2.3. Binning for Putative Genomes

Following the assembly of the metagenomes, the original reads were mapped back to the assembly in order to obtain a coverage, using the Burrows–Wheeler Aligner [30]. Subsequently, the coverage files, along with the final assembly, were binned for putative genomes using MetaBAT software [31]. Quality of the genomes, including completeness and contamination, was assessed using the CheckM tool [32]. The taxonomy of the quality bins obtained was assessed by means of amino acid identity (AAI), using the Microbial Genome Atlas (MiGA) web interphase [33]. Taxonomic novelty was determined by the maximum average amino acid identity (AAI) found against the genomes in the database. The p-value for this was estimated from the empirical distribution observed in all the reference genomes of NCBI’s RefSeq at each taxonomic level, and indicates the probability of the observed AAI between genomes in the same taxon. Phylogeny using AAI was also determined using MiGA. Trees were generated using iTOL software [34]. Annotation of all genomes was carried out using the Rapid Annotation using Subsystems Technology (RAST) pipeline [35].

## 3. Results and Discussion

### 3.1. Sampling Site Conditions and Sequencing Analysis

The area of study was the solar salterns of Cabo Rojo, Puerto Rico. They are located at the coordinates 17°57′25.2″ N, 67°11′58.0″ W, which represent the southwestern part of the Island (Figure 1). This artisanal solar saltern has 508 years of continous operation. Water from the Fraternidad Lagoon (salinity approximately 14–19% w/v) was pumped to the crystallizers and the evaporation cycle took approximately 60 days. Samplings were performed at the middle to the end of the evaporation cycle (between 35 and 45 days). The crystallizers sampled on the three different occasions exhibited an average temperature of 31.1 °C and a NaCl concentration of 34% (w/v).

After DNA sequencing, assessment for the quality of assembly for the metagenome was based on number of contigs, longest contig length, and N50 (the minimum contig length in the set of contigs that comprises over half of the assembly) (Table 1). A lower number of contigs and high contig length and N50 are ideal for high-quality assemblies [36]. Table 1 also details the assembly statistics using MetaQUAST [37] for the three metagenomes, where the millions of reads were condensed to one hundred thousand contigs, a significant reduction. Additionally, the N50 values obtained surpassed the values obtained in other metagenomic studies performed in hypersaline environments [21].

### 3.2. Microbial Community Composition

**Figure 2:**
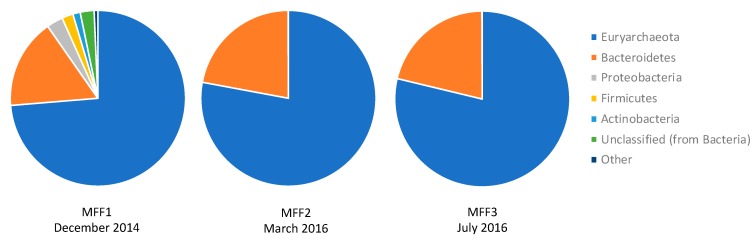
Taxonomic hits by phylum. Each slice indicates the number of reads with predicted 16SrRNA genes annotated to the indicated phylum. Samples were named MFF1 for the first sampling, MFF2 for the second sampling and MFF3 for the third sampling (MFF stands for “Muestra Final Fraternidad” which means Fraternidad Final Sample). Phylum *Euryarchaeota* were shown to be dominant in the three samples (73.69% for MFF1, 77.92% for MFF2, and 78.75% for MFF3), followed by *Bacteroidetes* (16.59, 22.03, 21.18%, respectively), and *Proteobacteria* (3.03, 0.05, 0.07%, respectively). Other groups include *Acidobacteria*, *Chlamydiae*, *Cyanobacteria*, *Deinococcus-Thermus*, *Fusobacteria*, *Planctomycetes*, and *Verrucomicrobia*, with less than 1% in MFF1.

The taxonomical assignment of the sequencing reads containing the 16S rRNA gene is shown in Figure 2. In terms of the microbial diversity present, the phylum Euryarchaeota predominated in all three metagenomes, with more than 70% of the reads. This was also observed when the analysis was performed with annotated reads with predicted proteins and ribosomal RNA genes (Figure S1). This abundance is expected, since this group contains the halophilic representatives from the Archaea domain. The Bacteroidetes group was the second in abundance, with about 20% of the reads. This group possesses one extremely halophilic representative in *Salinibacter ruber*. The third most predominant group was the Proteobacteria, with 1–3% of the reads. The presence of Proteobacteria was also expected, since it contains halophilic/halotolerant bacteria, such as the genera *Halomonas, Halovibrio*, and *Rhodovibrio*, among others [2]. Furthermore, taxonomic hits in MFF1 (as illustrated in Figure 2) reveal a diverse representation of other prokaryotic phyla. This representation is markedly different from the results found by Ghai et al. and Rhodes et al. [7,38], where only *Euryarchaeota*, *Bacteroidetes*, and *Proteobacteria* were encountered at a similar salinity of about 34% (w/v) or above. However, MFF2 and MFF3 were less diverse, with only three prokaryotic phyla detected, more consistent with the aforementioned results.

When assessing microbial diversity at genus level (Table 2), the community structure showed high stability, with the same three dominant genera (*Haloquadratum, Salinibacter, Halorubrum*) through time. However, variations in the diversity of less frequent genera were observed across all three metagenomes. Other studies performed in hypersaline environments, with a few notable exceptions [39,40], have demonstrated that *Haloquadratum* usually predominates in salinities of 30% (w/v) and higher [1,8,9]. Our study shows that *Haloquadratum* is the dominant genera at the solar salterns of Cabo Rojo, and the first to show the predominance of this genus through time in tropical environments. Podell et al., [41] demonstrated that *Haloquadratum* abundance was positively correlated with high levels of potassium, magnesium, and sulfide, and negatively correlated with an increase in microbial diversity. Ionic composition data obtained by Rodríguez-García [22] in the Cabo Rojo salterns on June 2015 (1.0 inches of precipitation in the area) showed high concentrations of chloride ions (230 g/L), followed by magnesium (28.84 g/L) and potassium (11.22 g/L), typical for a thalasohaline environment. These data show, as in other marine solar salterns around the world, that the ionic composition in these crystallizer ponds is suitable for *Haloquadratum* predominance. Ionic composition could change over time due to precipitation effects. Data obtained from the National Weather Service’s Advance Hydrologic Prediction Services (http://water.weather.gov/precip/) revealed that the amount of rainfall in the area of the Cabo Rojo salterns in November 2014 (first sampling was performed at the beginning of December 2014), March 2016, and July 2016 was 20.3 cm, 0.3 cm, and 7.6 cm of rain, respectively. These events were shown not to have an effect on salinity, as the salinity remained consistent at 34% (w/v) across all three samples, as well as the predominance of *Haloquadratum*. Precipitation is one of the many ways new microbes can be dispersed into new habitats [42]. Therefore, during a rain event, aquatic habitats can be recipients for new microorganisms. The data on precipitation suggest that the rainfall events in November could have contributed changes in ionic composition or a dilution effect on the water surface (causing cell lysis for haloarchaea) that directly affected microbial community structure and could explain the higher diversity of prokaryotic phyla in MFF1. Since Cabo Rojo is at a tropical location, Saharan dust could have also influenced precipitation and contributed to an increase in magnesium and potassium ions, which favored growth of *Haloquadratum* in all three samples [42]. Similarly, the amount of potassium found at this tropical saltern could favor an abundance of “salt-in” strategists, such as *Haloquadratum* and *Salinibacter*, and could perhaps be a contributing factor of their predominance in the three metagenomes.

Temporal studies in hypersaline environments at stable tropical climates are scarce. Most of these studies have been performed in variable climates, like the Lake Tebenquiche (Salar de Atacama) in Chile [43], and the Ocnei Lake in the Transylvanian Basin, Romania [44]. Even viruses have been the subject of temporal studies at solar salterns [45]. An interesting example of a temporal study performed at a less stable climate is the one at the Great Salt Lake in Utah, where the results showed that Salinibacter dominated as the main bacterial group through all samplings, whereas for the Archaea, Haloquadratum was also present in high numbers, although its abundance varied year-round. Other members of the haloarchaea found were *Halorubrum*, *Natronococcus*, and *Haloplanus*. The population changes observed in the Great Salt Lake were attributed to biotic factors, such as viruses and nutrients, and less to the seasonal temperature changes [46]. The presence of viruses could be a factor affecting microbial populations at salterns in tropical environments. Overall, the prokaryotic community structure of the crystallizers at the solar salterns in Cabo Rojo showed some variation at phylum-level but at genus-level seems to be stable over time.

### 3.3. Functional Annotation

In order to study the microbial processes that might be occurring in the Cabo Rojo solar salterns, contig sequences from the metagenomes were compared to the KEGG Orthology database and grouped into functional categories for further analysis.

**Figure 3:**
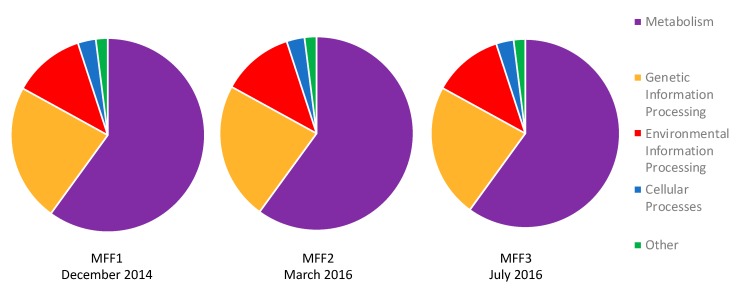
KEGG Orthology (KO) of functional genes obtained from samples MFF1, MFF2, and MFF3. Genes related to metabolism dominate in about 60% of the sequences in all three samples, followed by genetic information processing (23%), environmental information processing (12%), and cellular processes (3%). Other funtions are less than 1%. Samples were named MFF1 for the first sampling, MFF2 for the second sampling, and MFF3 for the third sampling (MFF stands for “Muestra Final Fraternidad” which means Fraternidad Final Sample).

Figure 3 details the predicted protein sequences of each metagenome into functional categories, according to the KEGG orthology database. Around 60% of the contigs obtained in all three samples were related to metabolism. This is consistent with other results in hypersaline environments, where a great number of metabolic processes, such as the primary production and degradation of organic compounds, are carried out [47]. Primary production is usually carried out at salinities of 25% NaCl and above, solely by the halophilic green algae *Dunaliella* [48,49]. However, it has been found that certain cyanobacteria are also capable of thriving at high salinities. For instance, cyanobacteria phylogenetically close to the genus *Halothece* have been reported in the Atacama Desert in Chile, with an average salinity of about 15% NaCl (w/v) [50]. Our results showed that cyanobacteria might be present at 34% NaCl in the crystallizers of Cabo Rojo. This was different from the Santa Pola salterns, where Ghai et al. reported that cyanobacteria were absent at salinities of 19% (w/v) and above [8].

**Figure 4:**
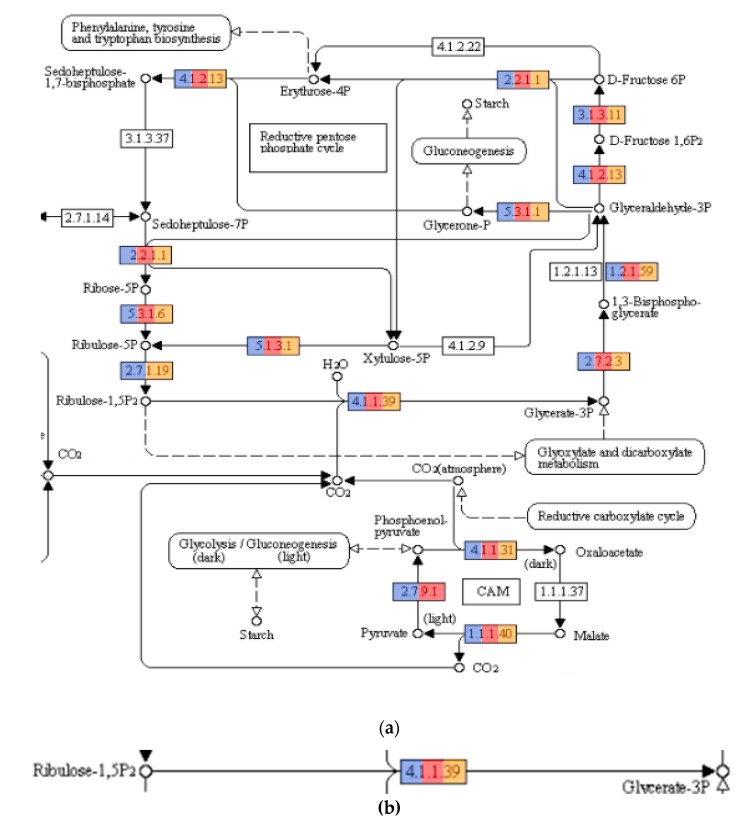
(**a**) Carbon metabolism pathways detected in the three metagenomes. Colored enzymes indicate the presence of the enzyme, light blue indicates presence in MFF1, red indicates presence in MFF2, and orange indicates presence in MFF3 (number of hits for each enzyme can be seen at Table S1 of supplementary figures). (**b**) Reaction catalyzed by the enzyme Ribulose-1,5-bisphosphate carboxylase/oxygenase (RuBisCO). The enzyme is present in all three metagenomes. Pathways obtained from KEGG pathways (*https://www.genome.jp/kegg/pathway.html*) with permission.

Genes related to carbon fixation were encountered in all three metagenomes (Figure 4). Particularly, the gene encoding for the Ribulose-1,5-bisphosphate carboxylase/oxygenase enzyme, more commonly known as RuBisCO, was present. This enzyme is critical for carbon fixation because it catalyzes the very first step in the Calvin cycle. Cyanobacteria carry out carbon fixation using RuBisCO in their carboxysomes. Analysis of the 16SrRNA gene hits revealed the presence of *Cyanobacteria* in MFF1 but in low numbers (0.01%). However, when looking for predicted protein sequences attributed to RuBisCO, we could not find any related to *Cyanobacteria* in the metagenomes. RuBisCO sequences were attributed to *Natronomonas* and *Halomicrobium*, both of which have been reported to possess this enzyme and have also been found in other metagenomic studies [51]. We were able to obtain almost complete genome bins from a putative new species of *Natronomonas* (Bin RC33) and of *Halomicrobium* (Bin RC39) from the metagenomes. These two putative species might have an important role in carbon fixation at the solar salterns of Cabo Rojo. However, transcriptomic approaches would be necessary in order to determine if the genes present in both species are metabolically active at a salinity of 34% (w/v).

**Figure 5:**
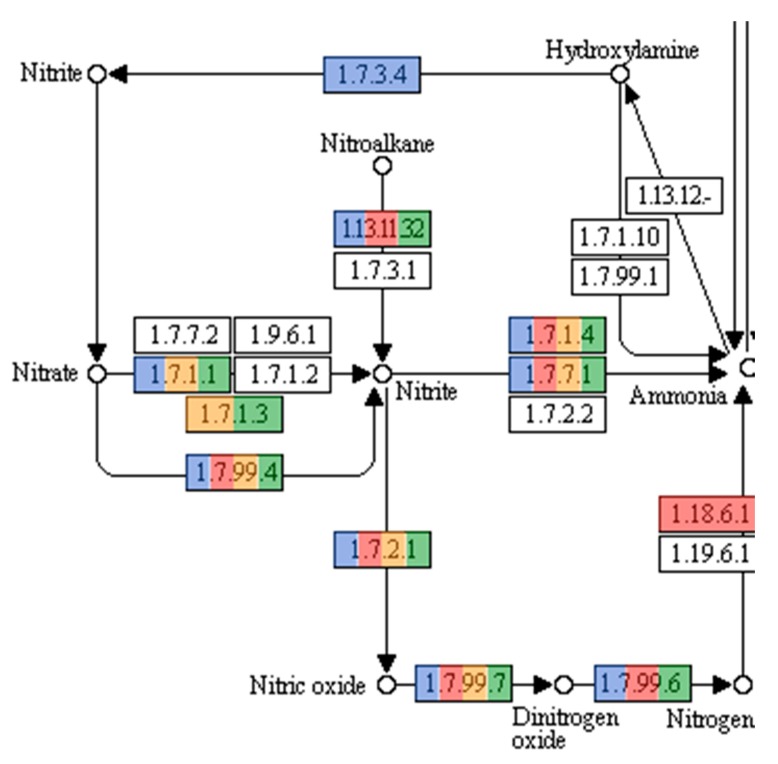
Nitrogen metabolism pathways present in the three metagenomes. Colored enzymes (Light blue and green for MFF1, red for MFF2, orange for MFF3) indicate the presence of hits related to the enzyme in the metagenome (number of hits for each enzyme can be seen at Table S1 of supplementary figures). Enzymes related to reductive pathways in the nitrogen cycle were encountered, including nitrate reductase (Enzyme Nomenclature database numbers (EC) 1.7.1.1, 1.7.1.3, 1.7.99.4), nitrite reductases (EC 1.7.2.1, 1.7.7.1, 1.7.1.4), and the nitrogenase needed for nitrogen fixation (EC 1.18.6.1). Pathways obtained from KEGG pathways (*https://www.genome.jp/kegg/pathway.html*) with permission.

Microorganisms also play a pivotal role in the nitrogen cycle. Bacteria, in particular, are involved in all N-cycle pathways and their nitrogen metabolism has been studied extensively [52]. However, nitrogen metabolism in archaea is not well studied or understood. Archaea are known to be participants in all the reductive pathways of the N-cycle. Archaea inhabiting extreme environments are considered the principal driving force of the N-cycle [52,53]. Figure 5 illustrates the pathways concerning the nitrogen cycle and, as expected, genes encoding for enzymes related to the reductive pathways of the N-cycle were present. Nitrogen fixation, the process catalyzed by a nitrogenase, in which atmospheric nitrogen is converted to ammonia, is performed naturally by both bacteria and archaea. In Archaea, nitrogen fixation has been reported in the methanogenic representatives of the phylum *Euryarchaeota* [53]. Methanogenic archaea were not detected in this study using 16S rRNA genes, however, reads with predicted protein sequences showed similiarities to functions related to nitrogen fixation.

Nitrification, the conversion of ammonia to nitrite and subsequently nitrate, is another pivotal process in the nitrogen cycle. Until recently, it was believed that this process was only undertaken by bacteria. However, several ammonia oxidizing archaea have been described, all representatives of the phylum *Thaumarchaeota* [54]. Protein sequences related to *Nitrosopumilus*, an ammonia oxidizing archaeon, were found in the three metagenomes [55,56]. However, as Figure 5 shows, there were no sequences matching the ammonia monooxygenase (AMO) enzyme. It has been argued that due to the high oxidation state of ammonia and the high energetic burden placed on halophilic organisms, this process would be too energy consuming for the amount of energy produced, and therefore not possible in high salinity environments [57]. This could suggest that either the sequences obtained are of an organism closely related to *Nitrosopumilus*, or that if the process is being performed, it could be the result of a novel less energy expensive pathway of ammonia oxidation. As sequencing technology improves, combined with better bioinformatics tools to analyze the enormous amounts of data, these gaps in information will be reduced.

**Figure 6:**
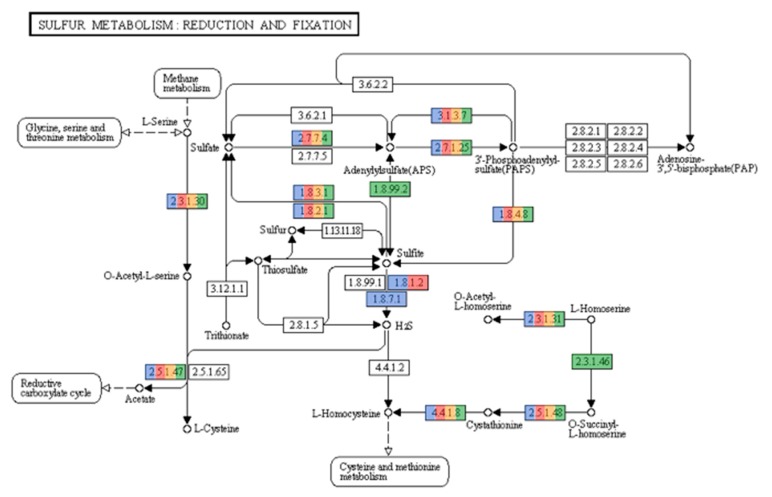
Sulfur metabolism pathways present in the metagenomes. Colored enzymes indicate the presence of hits related to the enzyme in the metagenome (number of hits for each enzyme can be seen at Table S1 of supplementary figures). Enzymes related to sulfate reduction (2.7.7.4, 1.8.99.2, 1.8.1.2, 1.8.7.1) were encountered. Pathways obtained from KEGG pathways (*https://www.genome.jp/kegg/pathway.html*) with permission.

As illustrated in Figure 6**,** hits matching sulfur metabolism were also found. The sulfur cycle is another prominent biogeochemical process undertaken in hypersaline environments. Both archaea and bacteria play a pivotal role in the cycling of sulfur. Sulfidogenesis, the production of H_2_S from the reduction of elemental Sulfur (S^0^), sulfate, thiosulfate, or sulfite, is a major step in the sulfur cycle. Bacteria possess sulfate reducing representatives in the phyla *Deltaproteobacteria* and *Firmicutes*. Sulfate-reducing bacteria in *Deltaproteobacteria* include the orders *Desulfobacteriales*, *Desulfovibrionales*, and *Syntrophobacteriales* [58], sequences of which were found in the three samples. Furthermore, the genus *Desulfotomaculum* from the *Firmicutes* was also present in our study. The archaeal genera known to reduce sulfate are *Archaeoglobus, Thermocladium*, and *Caldivirga*. However, hits related to these genera were not detected. This result is not surprising, because these organisms are not found at high salinity environments [59].

The oxidation of H_2_S is also another important pathway in the sulfur cycle, since hydrogen sulfide is toxic to plant and animal tissue. In hypersaline environments, representatives from the Gammaproteobacteria, such as *Halothiobacillus* and *Thiomicrospira*, among others, are classified as sulfur oxidizing bacteria (SOB) [60]. It is more common to find these types of bacteria in a hypersaline environment, due to the fact that their substrates are more reduced when compared to nitrifying organisms [57]. All three samples contained representatives from *Gammaproteobacteria*, including protein sequences matching those of *Halothiobacillus* and *Thiomicrospira*. Sulfur oxidizing archaea (SOA) have been poorly characterized and only two genera, Acidianus and Ferroglobus, are known to carry out sulfur oxidation [53]. Neither genus was encountered in our samples, nor were they expected, due to both being hyperthermophiles, growing optimally at temperatures above 60 °C.

These data show that microorganisms present at the solar salterns in Cabo Rojo might play an important role in the biogeochemical cycles, with most of the relevant pathways present in the metagenome. Furthermore, representatives known to perform processes in each of these cycles have been found. With further sampling, as well as the evolution of sequencing technologies, a more complete assessment can be carried out, as well as novel pathways being discovered that have not been described for the process at hypersaline environments [61,62].

### 3.4. Binning of Putative Novel Genomes

A reconstruction of putative genomes using binning techniques was performed, in order to determine if the predicted protein sequences found in the metagenomes could be assigned to specific organisms. Upon the assignment of taxonomic bins, it is important to avoid chimeric bins that might be produced which can lead to erroneous conclusions [19]. Caution should be taken before validating genomic bins, due to contaminating fragments. Of the software available, CheckM provides an accurate estimate of genome completeness and contamination [32]. A high number of taxonomic bins were obtained using binning methods, however most of these exhibited either a low degree of completeness or a high degree of completeness, but with high contamination. Nevertheless, we were able to obtain four genomic bins of significant quality from the three metagenomic libraries. All genomes presented a high amount of completeness and a low degree of contamination (Table 3).

Assigning taxonomy to uncultured organisms poses more of a challenge, due to the lack of phenotypic characterization. Therefore, the information available is based only on sequence data. The *Candidatus* status bypasses this limitation by assigning candidate names until phenotypic characters are appropriately characterized [63]. Several methods have been proposed to identify microbial species at the genome level. For cultured species, the DNA–DNA hybridization (DDH) has been the most traditional approach to differentiate closely related species with the 70% identity cutoff [64]. However, the average nucleotide identity (ANI) has been proposed as an alternate way of distinguishing bacterial and archaeal species. The cutoff for the ANI analyses is 95%, and has been employed successfully for the characterization of new microbial species [65,66,67]. Special caution should be taken, however, when describing species within a population, due to the members of a microbial population exhibiting gene differences of less than 5% of their total genes. Furthermore, ANI offers more robust resolution between genomes that share 80–100% ANI; organisms that show less than 80% ANI are too divergent to be compared based on this analysis [63]. Due to this, use of amino acid identity (AAI) is recommended to distinguish between more divergent organisms [66]. Organisms exhibiting an AAI of >85% are typically grouped within the same species, whereas those grouped in the genus-level exhibit an AAI of 60–80% [63,68]. Due to this, we used AAI to determine taxonomy for our four metagenomic bins (Table 4).

**Figure 7:**
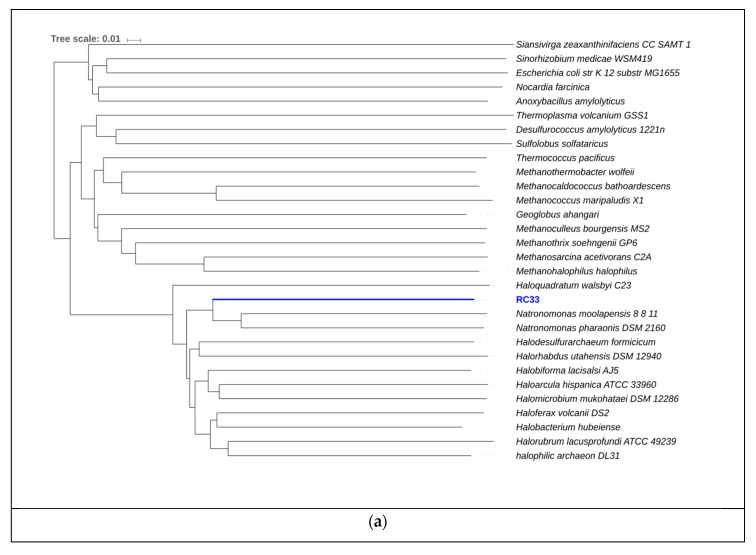

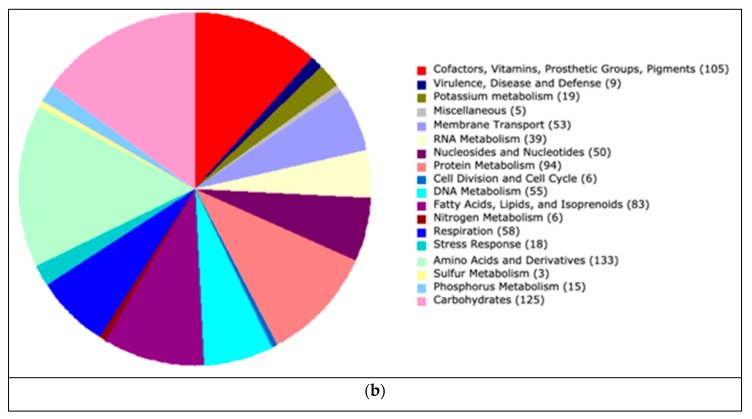
(**a**) Phylogeny for RC33 using amino acid identity. The scale represents change of amino acid substitution over time. (**b**) Subsystem category distribution for RC33. The graph represents the number of proteins that were grouped into a specific subsystems; 883 from a total 1570 coding sequences were identified to fit into subsystems. This chart was generated using Rapid Annotation System Technology (RAST).

AAI results for RC33 revealed that *Natronomonas moolapensis* is its closest relative, with an identity of 59.74%. Phylogeny using AAI (Figure 7) suggests that this genome is a member of the family Halobacteriaceae and could represent a new species within the genus (*p* value = 0.0038). The high GC content, as well as the presence of proteins associated with hyperosmotic stress, indicate that this organism is halophilic, as a characteristic of organisms thriving in hypersaline environments. The strain might be non-motile due to the absence of motility genes, gas vesicle clusters, and chemotaxis genes. Furthermore, several enzymes from the glycerol utilization cluster, including glycerol kinase and glycerol-3-phosphate dehydrogenase, were detected. The presence of these enzymes suggests that this strain could possibly grow on media containing glycerol. We attempted to identify if this organism could be associated with any of the biogeochemical pathways previously mentioned. We found hits associated with ammonia assimilation and reduction (E.C. 1.7.7.1 in Figure 5). However, no hits associated with significant steps in pathways of carbon fixation (Figure 4) and sulfur metabolism (Figure 6) were encountered.

**Figure 8:**
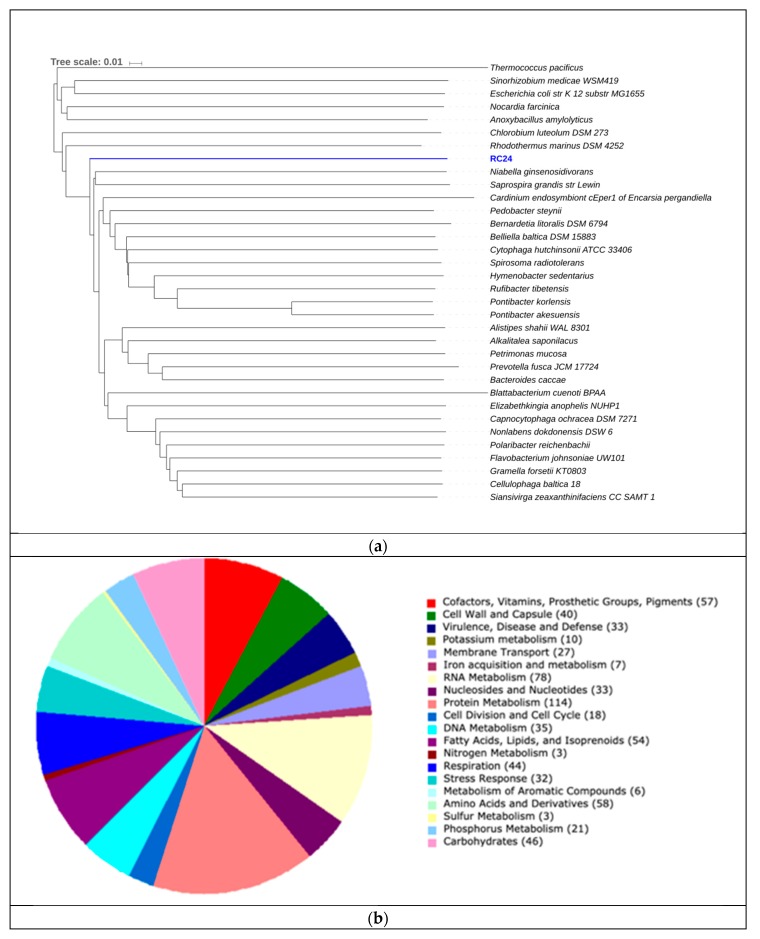
(**a**) Phylogeny for RC24 using AAI. The scale represents the number of amino acid substitutions over time. (**b**) Subsystem category distribution for RC24. The graph represents the number of proteins that were grouped into a specific subsystem; 723 of 1561 coding sequences were identified to fit into subsystems. This chart was generated using Rapid Annotation System Technology (RAST).

Bin RC24 showed close relatedness to *Pontibacter korlensis* (Figure 8). Proteins encoding gram-negative cell walls were matched in the genome, therefore classifying this organism as gram-negative. No chemotaxis proteins or flagellar proteins were found, suggesting that this bacterium was non-motile. Ammonia assimilation genes, including ammonium transporter and nitrite reductase (EC 1.7.1.4 in Figure 5) genes, were detected. We could not identify any significant genes related to carbon fixation pathways (Figure 4) or sulfur metabolism pathways (Figure 6). Due to its presence in high salinity, as well as its relatedness to *Pontibacter*, it is suggested to be halotolerant. Nevertheless, AAI, as well as statistical analyses performed in MiGA, suggest that this organism is a new genus of the Bacteroidetes (*p* value = 0.0021), and it is suggested that it could represent a new family within the phylum (*p* value = 0.0051). Its low GC content is unusual compared to other organisms in hypersaline environments, however Ghai et al. [8] obtained similar results when they described a new genus of low GC *Actinobacteria* in the Santa Pola salterns through binning methods.

**Figure 9:**
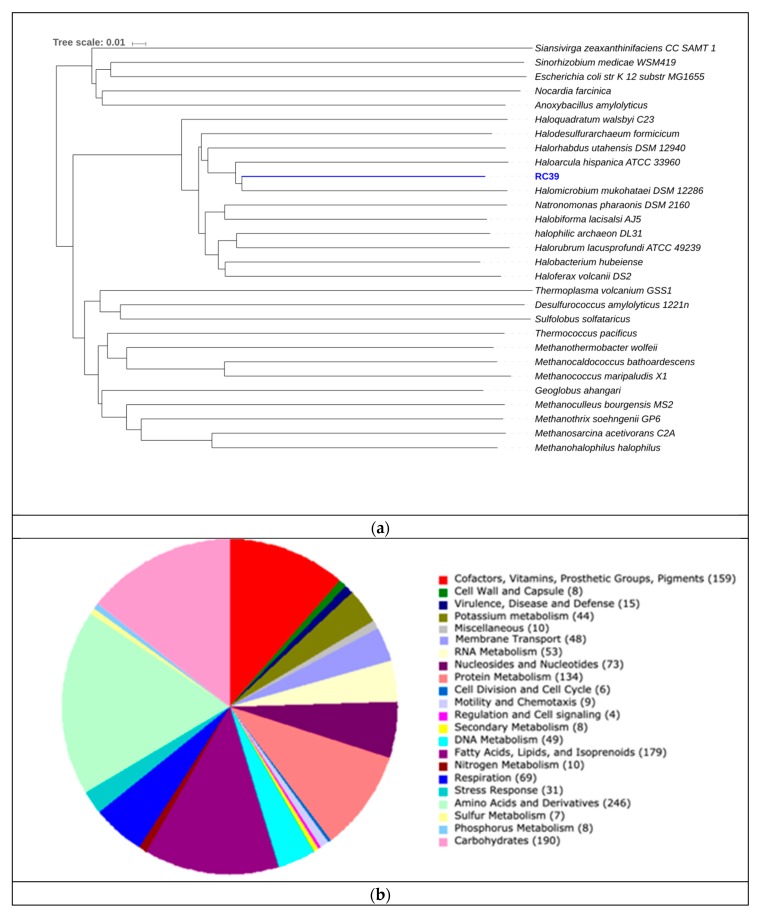
(**a**) Phylogeny for RC39 using AAI. The scale represents the number of amino acid substitutions over time. (**b**) Subsystem category distribution for RC39. The graph represents the number of proteins that were grouped into a specific subsystems; 1366 of 2439 coding sequences were identified to fit into subsystems. This chart was generated using Rapid Annotation System Technology (RAST).

RC39 showed 62.81% similarity *to Halomicrobium mukohataei* (Figure 9). The organism is motile, with genes encoding for archaeal flagellar proteins. Furthermore, this organism possesses the genes necessary for ammonia assimilation, as well as nitrate and nitrite reductases (EC 1.7.99.4, 1.7.7.1, 1.7.1.4 respectively in Figure 5). *Halomicrobium mukohataei* has been described to be able to grow anaerobically under the presence of nitrate, as a terminal electron acceptor and forming nitrite as an end product in anaerobic respiration [69]. Similar growth has also been observed in other organisms, such as *Corynebacterium glutamicum*, where nitrate was used as an electron acceptor, producing nitrite as an end product [70]. We could not identify any essential genes related to carbon fixation pathways (Figure 4) or sulfur metabolism pathways (Figure 6). Once again, the presence of glycerol kinases and glycerol-3-phosphate dihydrogenase suggest that this organism can grow on media containing glycerol. Our results suggest this organism to be a novel species of the genus *Halomicrobium* (*p* value = 0.0046).

**Figure 10:**
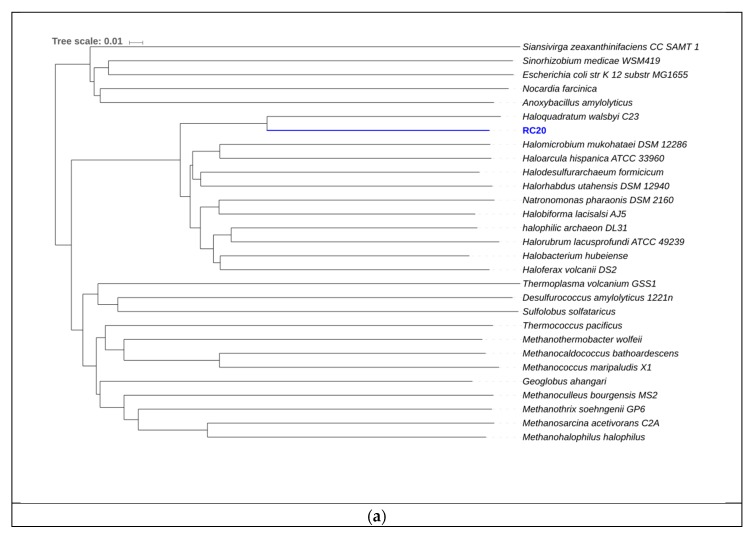

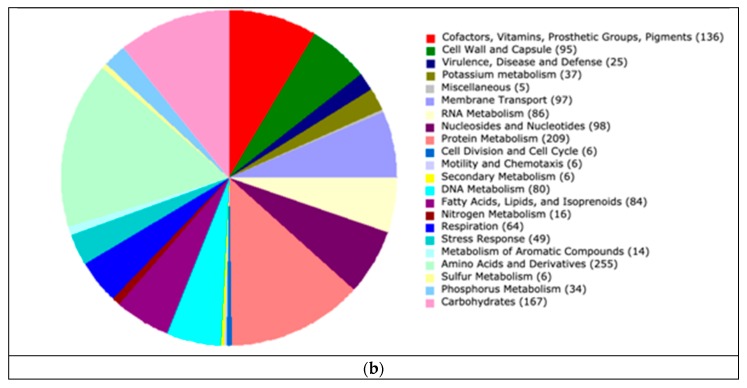
(**a**) Phylogeny for RC20 using AAI. The scale represents the amount of amino acid identity change over time. (**b**) Subsystem category distribution BIN20. The graph represents the number of proteins that were grouped into a specific subsystem; 1579 of 4980 coding sequences were identified to fit into subsystems. This chart was generated using Rapid Annotation System Technology (RAST).

Bin RC20 had an AAI of 65.83% with *Haloquadratum walsbyi* and MiGA analysis, suggesting it could represent a new species in the genus (*p* value = 0.0046) (Figure 10). The sequence of halomucin was blasted against the genome and was found. Halomucin, known as the largest archaeal protein, with 9159 amino acids, was described for the first time in *Haloquadratum walsbyi* [71]. Halomucin provides desiccation protection in saline environments to *Haloquadratum*, and is probably the secret to success for this organism in these environments. The presence of the gas vesicle cluster also coincides with the genome sequence of *Haloquadratum*. Bolhuis et al. [71] also described the presence of two bacteriorhodopsins and one halorhodopsin in *Haloquadratum*, which were also encountered here and is the reason they are able to grow phototrophically. This genome also encodes the presence of a TRAP (Tripartite ATP-independent periplasmic)-type C4-dicarboxylate transport system, two different ABC (ATP-Binding-Casette)-type sulfonate transport systems, and a phosphonate transport system, which are only described in *Haloquadratum walsbyi*. We detected genes related to nitrate/nitrite reductases (EC 1.7.99.4, 1.7.7.1, 1.7.1.4 in Figure 5). We also detected sulfate adenylyltransferase and adenylyl sulfate kinase (E.C. 2.7.7.4 and 2.7.1.25, respectively, in Figure 6) genes, both of which are important in sulfate reduction processes to sulfite [58]. No relevant genes related to carbon fixation pathways (Figure 4) were detected. The low GC content in this genome of 50.25% is also comparable to that of *Haloquadratum walsbyi* (47.9%). This low GC content is uncharacteristic of halophilic archaea due to their exposure to solar radiation. Due to the close relatedness of the genome with the *Haloquadratum walsbyi* genome, ANI was conducted in order to determine further resolution. ANI results showed an identity of 89.94%, indicating that RC20 is possibly a putative novel species of *Haloquadratum*.

Binning methods have uncovered previously undescribed microbes. As previously mentioned, Narasingarao et al. [20] recovered the recently proposed *Nanohaloarchaea* through binning methods. Furthermore, Ghai et al. [7] uncovered a novel group of low GC *Actinobacteria*, as well as a novel lineage of *Proteobacteria*, using metagenomic binning. Finally, through these methods, *Candidatus* “Nanopetramus” SG9 was also discovered [21]. Therefore, binning methods have been shown to be reliable in describing novel species in these environments, and may prove useful in providing a more non-biased assessment of the unculturable diversity in environmental samples. Moreover, a possible ecological role could be attributed to these reconstructed genomes. In our case, these putative genomes were shown to be associated to reductive pathways of the nitrogen cycle, as noted by the presence of nitrate and nitrite reductases in all four genomes. Additionally, we were able to identify a possible role of RC20 in sulfate reduction by the presence of related enzymes in sulfate adenylyltransferase and adenylyl sulfate kinase. One thing to note is that we were not able to detect any relevant genes related to carbon fixation, and it is therefore assumed that these organisms are not involved in these types of processes.

## 4. Conclusions

In this study, we used a deep sequencing strategy to obtain a considerable amount of sequence data (64 Gigabases) using a culture-independent approach, providing a more comprehensive perspective of microbial community structure and functional gene composition. By using a PCR unbiased culture-independent approach, a large microbial diversity has been discovered in the Cabo Rojo salterns. A diverse representation of prokaryotic phyla at a salinity of 34% (w/v) was shown, which has not been previously reported at this salinity. The microbial community structure at phylum-level could be influenced by weather fluctuations, which contribute to changes in the ionic composition of the crystallizer ponds. Additionally, using binning methods we have recovered four possible novel organisms that have been missed in our traditional culture-dependent surveys [12,13,14,15,16,17,18]. Although lack of phenotypic characterization of these strains makes validation of the organisms complicated, *Candidatus status* can be assigned to the four metagenomic bins. Further sampling may reveal more changes to this microbial community and can possibly unveil novel microbial groups. The importance of the organisms present in the Fraternidad crystallizer ponds is highlighted by the presence of essential genes related to the carbon, nitrogen, and sulfur cycles, with representatives from the diverse phyla encountered contributing to these cycles. Analysis of ionic composition of the crystallizer ponds following a precipitation event, as well as metatranscriptomics, may give us greater perspective regarding the active microbial community and their respective processes. Metagenomic analysis, although providing a wealth of information, does not distinguish active communities from dormant communities.

## Figures and Tables

**Table 1:** Sequencing results for the three metagenomes along with assembly statistics using MetaSPAdes assembler. Number of sequencing reads obtained through Illumina Sequencing, GC (Guanine Cytosine) content, range of read lengths are shown (in base pairs), number of assembled contigs, N50 and longest contig length are shown (in base pairs).

Sample	Number of Reads	GC Content (%)	Read Length (bp)	Number of contigs	N50 (bp)	Longest Contig (bp)
MFF1	29,432,758	56	35–251	318,469	3888	619,112
MFF2	41,746,817	57	35–151	420,402	4748	469,957
MFF3	40,634,465	58	35–151	379,415	4854	388,630

**Table 2:** Taxonomic composition at genus level for each metagenome, using 16SrRNA gene sequences. The percentage of reads aligning with a minimum length of 100 bp and 97% identity at genus level are shown. *Unclassified sequences belong to phylum Euryarchaeota.

MFF1		MFF2		MFF3	
Genus	Abundance	Genus	Abundance	Genus	Abundance
*Haloquadratum*	53.77%	*Haloquadratum*	69.76%	*Haloquadratum*	62.47%
*Salinibacter*	16.02%	*Salinibacter*	12.99%	*Salinibacter*	21.17%
*Halorubrum*	8.65%	*Halorubrum*	7.55%	*Halorubrum*	4.33%
Unclassified*	2.74%	*Halococcus*	1.99%	*Haloplanus*	4.12%
*Haloplanus*	2.39%	*Natronomonas*	1.39%	*Halococcus*	2.41%
*Haloarcula*	1.84%	*Halomicrobium*	1.08%	*Haloterrigena*	2.01%
*Pseudomonas*	1.74%	*Haloterrigena*	1.07%	*Natrinema*	1.08%
*Halococcus*	1.12%	*Haloplanus*	1.05%	*Natronomonas*	0.72%
*Natronomonas*	1.10%	*Haloferax*	0.45%	*Halovivax*	0.58%
*Haloferax*	0.94%	*Haloarcula*	0.43%	*Halobaculum*	0.51%
*Halovivax*	0.66%	*Halobaculum*	0.41%	*Haloferax*	0.35%
*Dyella*	0.61%	*Halobacterium*	0.41%	*Halomicrobium*	0.10%
*Ruminococcus*	0.46%	*Halogeometricum*	0.37%	*Haloarcula*	0.10%
**Total sequences**	19,228		37,156		31,202

**Table 3:** Details of genomic bins obtained.

Bin Name	Completeness (%)	Contamination (%)	GC Content (%)	Number of Contigs	N50 (bp)	Genome Size (Mb)	Predicted Proteins
RC33	94.40	2.40	66.22	11	392,903	1.5	1576
RC24	80.20	1.80	52.79	28	125,394	1.8	1533
RC39	96.00	3,20	68.18	75	54,228	2.4	2502
RC20	88.66	3.20	50.25	273	24,930	4.3	4449

**Table 4:** Amino acid identity (AAI) of the four genomic bins obtained. The closest relative of each organism, along with its AAI and fraction of proteins shared, are listed.

Bin	Closest Relative	AAI	Fraction of Proteins Shared
RC33	*Natronomonas moolapensis*	59.74%	81.85%
RC24	*Pontibacter korlensis*	43.31%	66.02%
RC39	*Halomicrobium mukohataei*	62.81%	68.27%
RC20	*Haloquadratum walsbyi*	65.83%	73.46%

## Supplementary Materials

All data can be found at the NCBI Sequence Read Archive under BioProject PRJNA343765 under accession numbers SRR8816317, SRR8816318 and SRR8816319 for samples MFF1, MFF2 and MFF3 respectively. The following are available online at https://www.mdpi.com/2073-4425/10/6/422/s1, Figure S1: Taxonomic hits by phylum of assembled contigs, Table S1: Kegg pathway hits for nitrogen metabolism, photosynthesis, and sulfur metabolism obtained from metagenomes MFF1, MFF2, and MFF3.
