# Supplementary material for: Temporal Analysis of the Microbial Community from the Crystallizer Ponds in Cabo Rojo, Puerto Rico, Using Metagenomics
Source: Genes (Basel). 2019 May 31;10(6):422. doi: 10.3390/genes10060422 (PMC6627146; doi:10.3390/genes10060422)
Supplement: Supplementary file 1 [file genes-10-00422-s001.pdf]

Supplementary Figures

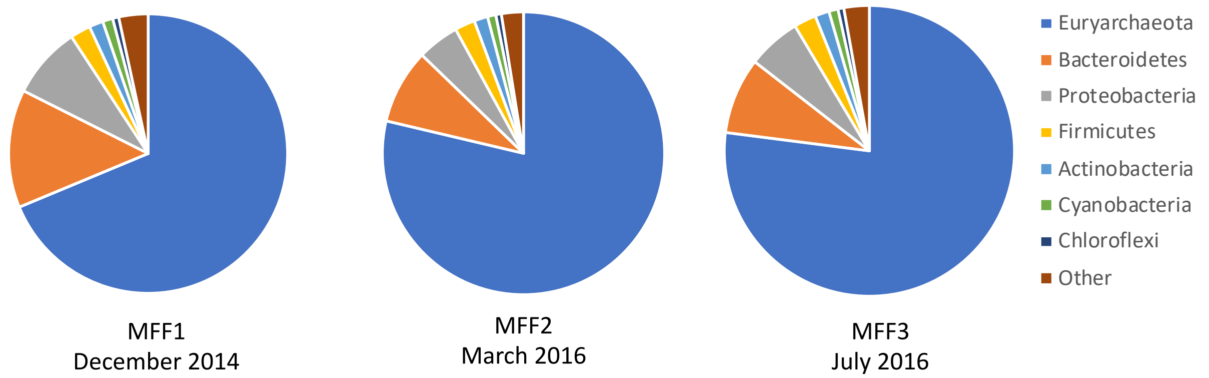

Figure S1. Taxonomic hits by phylum of assembled contigs. Each slice indicates number of reads with predicted proteins and ribosomal RNA genes annotated to the indicated phylum. Samples were named MFF1 for the first sampling, MFF2 for the second sampling and MFF3 for the third sampling (MFF stands for "Muestra Final Fraternidad" which means Fraternidad Final Sample). Phylum *Euryarchaeota* showed to be dominat in the three samples (68.74% for MFF1, 78.69% for MFF2, and 77.02% for MFF3) followed by *Bacteriodetes* (13.68, 8.54, 8.48% respectively), *Proteobacteria* (8.32, 4.78, 5.94% respectively), *Firmicutes* (2.36, 2.32, 2.47% respectively), *Actinobacteria* (1.62, 1.57, 1.58% respectively). *Cyanobacteria* and *Chloroflexi* were 1% or less in abundance on all metagenomes. Other groups (3%) include, *Crenarchaota, Korarchaeota, Nanoarchaeota, Thaumarchaeota, Aquificae, Deinococcus-Thermus* and *Nitrospirae*.

Table S1. KEGG pathway hits for nitrogen metabolism, photosynthesis, and sulfur metabolism obtained from metagenomes MFF1, MFF2, and MFF3.

KEGG Pathway	Function	MFF1	MFF2	MFF3
00910 Nitrogen metabolism [PATH:ko00910]	nitronate monooxygenase [EC:1.13.12.16]	1	0	1
00910 Nitrogen metabolism [PATH:ko00910]	nitrate reductase (NADH) [EC:1.7.1.1]	1	1	1
00910 Nitrogen metabolism [PATH:ko00910]	nitrite reductase (NO-forming) [EC:1.7.2.1]	10	31	23
00910 Nitrogen metabolism [PATH:ko00910]	nitrate reductase catalytic subunit [EC:1.7.99.4]	41	79	75
00910 Nitrogen metabolism [PATH:ko00910]	NIAD; nitrate reductase (NADPH) [EC:1.7.1.3]	0	2	1

00910 Nitrogen metabolism [PATH:ko00910]	cah; carbonic anhydrase [EC:4.2.1.1]	9	19	13
00910 Nitrogen metabolism [PATH:ko00910]	cynT, can; carbonic anhydrase [EC:4.2.1.1]	70	91	66
00910 Nitrogen metabolism [PATH:ko00910]	hao; hydroxylamine oxidase [EC:1.7.3.4]	2	0	0
00910 Nitrogen metabolism [PATH:ko00910]	narZ; nitrate reductase 2, alpha subunit [EC:1.7.99.4]	2	0	0
00910 Nitrogen metabolism [PATH:ko00910]	nifD; nitrogenase molybdenum-iron protein alpha chain [EC:1.18.6.1]	2	0	0
00910 Nitrogen metabolism [PATH:ko00910]	nifW; nitrogenase-stabilizing/protective protein	1	0	0
00910 Nitrogen metabolism [PATH:ko00910]	nirA; ferredoxin-nitrite reductase [EC:1.7.7.1]	96	146	137
00910 Nitrogen metabolism [PATH:ko00910]	nirB; nitrite reductase (NAD(P)H) large subunit [EC:1.7.1.4]	2	2	2
00910 Nitrogen metabolism [PATH:ko00910]	nirD; nitrite reductase (NAD(P)H) small subunit [EC:1.7.1.4]	3	3	6
00910 Nitrogen metabolism [PATH:ko00910]	norB; nitric oxide reductase subunit B [EC:1.7.2.5]	6	37	26
00910 Nitrogen metabolism [PATH:ko00910]	norC; nitric-oxide reductase, cytochrome c-containing subunit II [EC:1.7.99.7]	1	0	0
00910 Nitrogen metabolism [PATH:ko00910]	norF; nitric-oxide reductase NorF protein [EC:1.7.99.7]	7	3	8

00910 Nitrogen metabolism [PATH:ko00910]	nosZ; nitrous-oxide reductase [EC:1.7.2.4]	4	0	13
00910 Nitrogen metabolism [PATH:ko00910]	nrfC; protein NrfC	1	1	0
00910 Nitrogen metabolism [PATH:ko00910]	nrfD; formate-dependent nitrate reductase complex, transmembrane protein	0	0	1
00710 Carbon fixation in photosynthetic organisms [PATH:ko00710]	rbcL; ribulose-bisphosphate carboxylase large chain [EC:4.1.1.39]	13	17	13
00920 Sulfur metabolism [PATH:ko00920]	sulfite dehydrogenase [EC:1.8.2.1]	1	0	0
00920 Sulfur metabolism [PATH:ko00920]	SUOX; sulfite oxidase [EC:1.8.3.1]	5	1	3
00920 Sulfur metabolism [PATH:ko00920]	met3; sulfate adenylyltransferase [EC:2.7.7.4]	84	120	124
00920 Sulfur metabolism [PATH:ko00920]	cysQ, MET22, BPNT1; 3'(2'), 5'-bisphosphate nucleotidase [EC:3.1.3.7]	9	5	3
00920 Sulfur metabolism [PATH:ko00920]	aprB; adenylylsulfate reductase, subunit B [EC:1.8.99.2]	0	0	2
00920 Sulfur metabolism [PATH:ko00920]	cysC; adenylylsulfate kinase [EC:2.7.1.25]	23	26	28
00920 Sulfur metabolism [PATH:ko00920]	cysD; sulfate adenylyltransferase subunit 2 [EC:2.7.7.4]	30	23	12

00920 Sulfur metabolism [PATH:ko00920]	cysH; phosphoadenosine phosphosulfate reductase [EC:1.8.4.8]	141	194	235
00920 Sulfur metabolism [PATH:ko00920]	cysJ; sulfite reductase (NADPH) flavoprotein alpha-component [EC:1.8.1.2]	2	0	0
00920 Sulfur metabolism [PATH:ko00920]	cysN; sulfate adenylyltransferase subunit 1 [EC:2.7.7.4]	6	4	5
00920 Sulfur metabolism [PATH:ko00920]	cysNC; bifunctional enzyme CysN/CysC [EC:2.7.7.4 2.7.1.25]	52	27	15
00920 Sulfur metabolism [PATH:ko00920]	sir; sulfite reductase (ferredoxin) [EC:1.8.7.1]	1	0	0
